# Selective inhibition of NikA mediated Ni(II) import in *E. coli* by the Indium(III)-EDTA complex

**DOI:** 10.1093/mtomcs/mfaf008

**Published:** 2025-03-04

**Authors:** Stephanie Sebastiampillai, Mark Nitz

**Affiliations:** Department of Chemistry, University of Toronto, Toronto, Ontario, M5S 3H6, Canada; Department of Chemistry, University of Toronto, Toronto, Ontario, M5S 3H6, Canada

## Abstract

Nickel is a required nutrient for bacteria to produce [NiFe]-hydrogenase and urease enzymes. [NiFe]-hydrogenase catalyzes the reversible conversion of hydrogen into protons and electrons and urease catalyzes the hydrolysis of urea into carbon dioxide and ammonia—both key in bacterial pathogenesis. As such, nickel trafficking and homeostasis are interesting targets for potential antibacterial strategies. In *E. coli*, NikA binds a Ni(II)-(L-His)_2_ chelate in the periplasm and delivers this complex to the NikBCDE transporter. Blocking Ni(II) uptake by NikA would prevent the biosynthesis of active [NiFe]-hydrogenase. Fe(III)-EDTA is a potent ligand for NikA, however due to the potential for reduction of Fe(III) to Fe(II), it has limited utility. Using Fe(III)-EDTA as a starting point for inhibitor design, similar stable complexes of Bismuth(III), Lutetium(III) and Indium(III) were investigated. The In(III)-EDTA complex is a potent inhibitor of cellular [NiFe]-hydrogenase activity (IC_50_ of 600 μM ± 100 μM) while being nontoxic to bacterial growth. The mechanism of In(III)-EDTA hydrogenase inhibition was confirmed by the inhibition of Ni(II)-dependent processing of HycE (hydrogenase-3), which could be rescued with the addition of exogenous nickel. To elucidate the binding affinity of In(III)-EDTA to NikA, isothermal titration calorimetry (ITC) was carried out, revealing stoichiometric 1:1 binding with a *K*_d_ of 17.3 µM ± 3.0 µM. Indium concentrations determined by inductively coupled plasma mass spectrometry in *E. coli* cells in the presence or absence of NikA showed no discernable difference, further supporting the competitive inhibition of nickel uptake by blocking NikA.

## Graphical Abstract

**Graphical Abstract:**
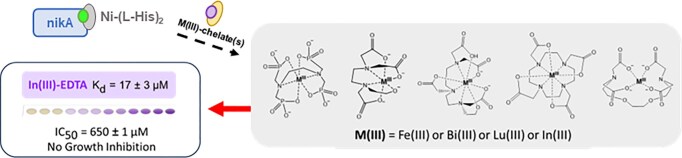

## Introduction

Nickel is found in key enzymes of plants, bacteria, and archaea but has no known role in human biology [1–4]. Of the bacterial nickel-dependent enzymes, urease and [NiFe]-hydrogenase are of particular interest due to their pivotal role in survival and pathogenesis. Urease requires nickel in its active site to enable the breakdown of urea [5–7]. Similarly, [NiFe]-hydrogenases require nickel in its bimetallic center to catalyze the reversible, production or consumption of hydrogen [8–10]. Common gut infecting pathogens such as *Salmonella typhimurium* [11] and *Campylobacter jejuni* [12] require active [NiFe]-hydrogenase to maintain growth, colonization, and acid resistance; whereas, uropathogenic *Escherichia coli* [13] requires [NiFe]-hydrogenase for acid resistance and maintaining a proton motive force [14]. S*taphylococcus aureus* and shiga toxin producing *E. coli* use urease for their acid response and to improve host colonization [7, 14, 15]. Alternatively, pathogens such as *Mycobacterium tuberculosis, Actinobacillus pleuropneumonia, Klebsiella pneumoniae*, and *Helicobacter pylori* require both urease and [NiFe]-hydrogenases for successful virulence and targeting either or both enzymes results in attenuation of key pathogenesis [6, 16–23].

As nickel is usually found at low concentrations in the environment, bacteria use a collection of nickel metallochaperones to control nickel uptake and movement [2]. Inhibiting the nickel biosynthetic pathway is a promising strategy for developing antibacterials [8, 24]. Additionally, interrupting bacterial nickel homeostasis is a known antibacterial strategy used by humans, as seen with the protein calprotectin that sequesters nickel from infecting pathogens [25, 26]. Here, we use [NiFe]-hydrogenase, the main nickel-based enzyme found in our *E. coli* model, as the terminus of the nickel import pathway to monitor the activity of pathway inhibitors.

In *E. coli*, nickel(II) import begins with the binding of a Ni(II) complex to the periplasmic protein, NikA [27, 28]. Studies performed with *E. coli* knockout strains of NikA show decreased hydrogenase activity, which could only be restored with the addition of exogenous nickel [29]. Molecules targeting NikA with high affinity, in theory, could starve the cells of nickel, blocking [NiFe]-hydrogenase maturation. The likely subsequent steps involve NikA-Ni(II) complex binding to the ABC-type NikBCDE transporter, leading to transport of Ni(II) into the cytosol. The mechanism of nickel transport through NikBCDE is not well understood, however, this process is coupled with the hydrolysis of ATP, which is hypothesized to occur at the NikD and NikE interface [7, 30, 31]. Once the nickel is in the cytosol, there are three main nickel metallochaperone proteins: HypA, HypB, and SlyD that sequester and escort nickel to the bimetallic catalytic center of [NiFe]-hydrogenase. The sequential movement of nickel is suggested to begin with nickel being transferred from NikE to HypB, with SlyD serving as a modulator and storage site for nickel as it is passed through the NikBCDE transporter [32, 33]. HypB is a weak GTPase, that requires GTP hydrolysis to initiate nickel transfer to HypA in its GDP bound state [32, 34, 35]. Through pulldown assays it has been shown that the HypB-HypA complex is bound to the large subunit of hydrogenase-3, the main hydrogenase responsible for H_2_ generation in conjunction with the formate hydrogenlyase complex found on the cytoplasmic side of the membrane [36–40]. Once nickel is inserted into the large hydrogenase subunit, it is coordinated by two thiolates and two bridging sulfur groups between the nickel and the iron found in the catalytic center [8, 24, 41]. This subsequently results in cleavage of a 32-residue C-terminal peptide of the large subunit, followed by the small subunit docking and the internalization of the [NiFe] center [8, 42, 43]. Monitoring final protease cleavage of HycE provides a convenient measure of [NiFe]-hydrogenase maturation as this does not occur prior to formation of the [NiFe] center.

The nickel pathway leading to [NiFe]-hydrogenase maturation is an interesting target for inhibition in many pathogens. Several small molecules have been shown to inhibit [NiFe]-hydrogenases, such as O_2_, CO, NO, or acetylene, which have been studied in the context of biomass-generated synthetic gas with [NiFe]-hydrogenase as the primary source of H_2_ in renewable fuel production [44]. Interaction of the aforementioned small molecules is complicated and in many cases pleiotropic, with multistep inhibition processes [24, 45]. Monitoring [NiFe]-hydrogenase activity allows discovery of inhibitors that target the nickel pathway. In our previous work, we screened a library of bioactive molecules and found that iodoquinol inhibits the pathway by sequestration of nickel in the environment, albeit iodoquinol is toxic to *E. coli* [17, 29].

In this study we focus on competitively inhibiting the first step of Ni(II) import by targeting extracellular Ni(II) binding. Phylogenetic analysis of bacterial extracellular Ni(II) binding proteins has shown at least 13 different clusters [46]. Structural and biochemical analysis of some of these proteins has shown that, at minimum, there are four different mechanisms of Ni(II) recognition [46–48]. These include direct recognition of Ni(II) (*Campylobacter jejuni* NikZ) [46, 49], recognition of Ni(II)His-thiazolidine (*Staphylococcus aureus* NikA) [48] and the recognition of Ni(II)-(L-His)_2_ by multiple mechanisms [46]. These different mechanisms of Ni(II) recognition provide a path for selectively inhibiting Ni(II) import by specific groups of bacteria. Here, we target *E. coli* NikA (*Ec*NikA), which recognizes Ni(II)-(L-His)_2_ [50]. The Ni(II) recognition mechanism of *Ec*NikA has been found to be conserved in alpha and gammaproteobacteria, including the pathogens *Klebsiella pneumoniae* [51], and *Brucella sui*s [46].

Recently, Sychantha *et al*. found that using the fungal metallophore, aspergillomarasmine (AMA) chelated to zinc(II) or cobalt(II) results in the inhibition of the urease and [NiFe]-hydrogenase activity in *Klebsiella pneumoniae* via Zn-AMA's tight binding to NikA (*K*_d_ = 6.8 μM) [51]. We take a similar approach to targeting [NiFe]-hydrogenase maturation. In this work, we use a whole-cell hydrogenase assay and hydrogenase maturation to study the inhibition of Ni(II) uptake by targeting *Ec*NikA [17]. Based on the known tight binding of Fe(III)-EDTA(H_2_O)^−^ to NikA [52], alternative chelate based inhibitors were explored. In(III)-EDTA was found to be potent in [NiFe]-hydrogenase inhibition and recovery assays, and through immunoblot analysis, was found to prevent [NiFe]-hydrogenase maturation.

## Experimental

### Metal(III)-chelator complex preparation

Chelator complexes of Iron(III), Lutetium(III), Bismuth(III), or Indium(III) were prepared as described below. Iron(III)-ethylenediaminetetraacetic acid (EDTA) complex was made by dissolving FeCl_3_ (Millipore Sigma) in a stock solution of EDTA (pH corrected with 1 M NaOH) at pH 8 at 1:1 equivalence to give a final concentration of 15 mM Fe(III)-EDTA. Lutetium(III), Bismuth(III), and Indium(III) complexed with EDTA were made as follows: the chloride salt of each metal(III) (Millipore Sigma) was dissolved into a buffer containing 15 mM EDTA (pH 8, corrected with 1 M NaOH, BioShop) and 50 mM sodium acetate (pH 8) to give a final metal(III)-EDTA concentration of 15 mM. The solutions of Lu(III)-EDTA, Bi(III)-EDTA, and In(III)-EDTA were mixed for about 15 min and allowed to clear. Approximately 15 mM of InCl_3_ was complexed with 15 mM of ethylene glycol-bis(β-aminoethyl ether)-N, N, N′, N′-tetraacetic acid (EGTA), N, N, N′, N′-ethylenediaminetetrakis(methylenephosphonic acid) (EDTPA), diethylenetriaminepentaacetic acid (DTPA), or dodecane tetraacetic acid (DOTA) at pH 8 and 50 mM sodium acetate, pH 8, the In(III)-DOTA complex was heated to 80°C for 20 min as described previously [53]. All complexes were filter sterilized with a 0.45 μm syringe filter once the solution became clear and completely dissolved.

### Whole-cell hydrogenase assay

The whole-cell hydrogenase assay was performed as previously described [17, 29]. Briefly, into a Corning Costar round bottom polystyrene 96 well plate (catalog number 3799) modified Tryptone-Yeast Extract-Tris (TYET, 10×) media was added containing a final concentration of 10 g/l tryptone (Bioshop), 5 g/l yeast extract (Bioshop), and 50 mM Tris (Bioshop), pH 7.5, with 0.4% glucose (Bioshop), 30 mM sodium formate (Sigma), 1 μM sodium selenite (Sigma), and 1 μM sodium molybdate (Sigma), inoculated with 0.8% (v/v) of an overnight culture incubated at 37°C with shaking of BW25113 *E. coli* to give a 200 μl final volume in each well. If the well was supplemented with metal(III)-chelators, nickel(II) sulfate or deionized water, the concentrations were adjusted so the final TYET concentration were as described above. The plates were covered with the supplied lid and incubated, microanaerobically, for 6 h at 37°C. Once the plates were cooled, the optical density (OD) at 630 nm was recorded with a Synergy H1 (BioTek) microplate reader. After the addition of the developing solution (10 mg/ml of benzyl viologen and 250 mM sodium formate in 20 mM Tris buffer, pH 7.5) into each sample well (20 μl), the change in absorbance was monitored every 30 s for a total of 5 min using the same microplate reader at ambient temperature.

### Metal chelate compatibility with the hydrogenase assay

Interference with the benzyl viologen reduction by the metal chelates was evaluated to ensure that any signal inhibition was not due to the metal(III)-chelator being reduced or by directly inhibiting the hydrogenase activity. This was done by growing the *E. coli* cells as described for the whole-cell hydrogenase assay, however, the cells were supplemented with the metal(III) chelate after they had been grown for 6 h at 37°C, immediately prior to assaying. The metal(III)-chelate was added to the wells (10−15 μl) and the colormetric change in benzyl viologen reduction was measured immediately. If attenuated signal was observed, this was indicative of the added metal(III)-chelate suppressing benzyl viologen reduction signal and thus directly interfering with the assay, as mature active [NiFe]-hydrogenase is present in these samples.

### Inhibition and recovery assays

The inhibition and toxicity of BW25113 *E. coli* cells was assessed by recording the reduction rates of benzyl viologen in the presence of In(III)-EDTA such that the concentration that caused 50% growth inhibition (GI_50_) and 50% hydrogenase activity inhibition (IC_50_) could be determined. The dose-response curves were fit to Equation 1. The benzyl viologen rates were divided by the OD_630_ measurement to normalize cell growth in each sample. The recovery assays were carried out by the addition of 1 mM NiSO_4_ before culture incubation with the respective concentration of the metal(III)-chelate.


(1) \begin{eqnarray*}
\frac{{\rm R}_{\rm x} - {\rm R}_{\rm min}}{{\rm R}_{\rm max} - {\rm R}_{\rm min}} = \frac{1}{1 + \left(\frac{{\rm X}}{{\rm X}_{\rm O}} \right)^{\rm n}}
\end{eqnarray*}

*R*_x_ is the response specific concentration *x. R*_min_ and *R*_max_ are the initial and final activities that are experimentally determined (representative of % response). The *x*_o_ is where half of the effect is seen [29].

### HycE immunoblotting

HycE immunoblots were performed as described previously [17]. BW25113 *E. coli* cells were plated on antibiotic-free Lennox broth (LB)/agar plates from a glycerol stock (−80°C). The *E. coli* cells were grown at 37°C without shaking overnight in LB media. Approximately 0.7% (v/v) of the overnight culture was added to a full sterile 50-ml falcon tube (∼57 ml) containing modified TYET media with or without the metal(III)-chelates with the same final concentrations as used in the whole-cell hydrogenase assay. The tube was capped, and the cells were grown anaerobically for 6 h at 37°C without shaking. Cells were harvested by centrifugation at 4000 rpm and washed once with ice-cold 50 mM Tris, pH 7.5 buffer. The cells were then resuspended into the ice-cold 50 mM Tris, pH 7.5 buffer containing 200 μM phenylmethylsulfonyl fluoride and 1 mM 1,4-dithiothreitol and were sonicated on ice (30 s on and 30 s off for three cycles). The sonicated lysate was cleared by centrifugation at 21.1× *g* and was stored at −80°C if not used immediately. Alongside the MagicMark XP protein standard and SeeBlue standard (Thermo), the protein samples were resolved on an 8% Sodium Dodecyl Sulfate-Polyacrylamide Gel Electrophoresis) SDS-PAGE) gel and transferred onto a polyvinylidene difluoride membrane (Millipore) and probed using a polyclonal anti-HycE antiserum (CedarLane) in a 1:1000 dilution. A secondary goat antirabbit antibody (Bio-Rad) was used in a 1:30 000 dilution. The bands were visualized using the SuperSignal West Pico Chemiluminescent Substrate Kit (Thermo).

### Protein expression and purification

The pET24b-*nikA* was described by W.W.H. Law [30, 54]. The plasmid was transformed into chemically competent BL21(DE3) *E. coli* cells and plated onto LB agar plates that contained 50 μg/ml of kanamycin, followed by incubation at 37°C. A single colony from the plate was used to inoculate 50 ml of LB containing 50 μg/ml of kanamycin and incubated overnight at 37°C while shaking. This overnight culture was then used to inoculate 4 l of LB media containing 50 μg/ml of kanamycin and grown to an OD_600_ of 0.6–0.8. Overexpression of NikA proteins was done with the addition of 0.2 mM IPTG and incubation overnight at 16°C with shaking.

The cells were harvested by sucrose osmotic shock as described previously. Firstly, the cells were resuspended in 500 ml of osmotic shock buffer [30 mM Tris-HCl, pH 8, 20% (w/v) sucrose] with 1 ml of 500 mM EDTA added dropwise while stirring at 4°C. This was left to stir for an additional 10 min at 4°C after all the EDTA had been added. The cells were pelleted at 3450× *g* at 4°C for 40 min. The pellet was gently resuspended in 100 ml of 5 mM MgSO_4_ and stirred at 4°C for 10 min. The periplasmic contents were separated from the spheroplasts by ultracentrifugation at 80 696× *g* at 4°C for 30 min. The supernatant was collected and 500 mM HEPES, pH 7.5 was added, so the final protein buffer concentration was 20 mM HEPES, pH 7.5.

The collected supernatant was loaded onto a diethylaminoethyl sepharose (GE Healthcare) column that was pre-equilibrated with 20 mM HEPES, pH 7.5. The NikA protein was eluted using a linear NaCl gradient of 0–200 mM, where the eluent fractions containing the NikA protein were pooled and dialyzed in 20 mM HEPES, pH 7.5 overnight at 4°C. The dialyzed fractions were then loaded onto the UnoQ (Bio-Rad), a strong anionic exchange column and the NikA proteins were eluted with a NaCl concentration of 30–80 mM. Purity of the NikA protein was confirmed by SDS-PAGE and electrospray ionization-mass spectrometry (ESI MS). ESI MS was performed at the Advanced Instrumentation for Molecular Structure Facility in the Department of Chemistry, University of Toronto, Toronto, ON. The mass of the wild-type NikA protein is 56 304 Da. The purified NikA protein was exchanged into the NikA buffer (20 mM HEPES, pH 7.5, 100 mM NaCl). Protein concentration was determined by measuring A_280_ with an extinction coefficient of 77 810 M^−1^cm^−1^.

### Isothermal titration calorimetry

A MicroCal Auto-iTC_200_ (Malvern) was used to conduct isothermal titration calorimetry (ITC) experiments. The wild type NikA protein was concentrated using a 3000 Da MWCO Amicon Ultra Centrifugal Filter (Millipore Sigma) to 250–350 μM. The stocks of Fe(III)-EDTA, In(III)-EDTA, and other In(III)-chelates were diluted to 2.5–3.5 mM in the degassed NikA buffer. ITC experiments were performed at 298 K with 20–25 injections of 2 μl of the respective ligand to the NikA protein solution in the sample cell (approximately 180 s between each 4 s injection). Control experiments were done by titrating the respective metal(III)-chelate(s) into degassed NikA buffer. The data was processed with the Origin software package (MicroCal), where the thermodynamic parameters were determined by fitting the ITC isotherms using a nonlinear least square minimized algorithm to a one site-binding model [30].

### ICP-MS

Indium(III) concentrations were determined using inductively coupled plasma mass spectrometry (ICP-MS). Samples were prepared as described in the HycE Immunoblotting procedure and harvested. The cells were resuspended in the ICP-MS buffer (200 mM Tris, pH 7.5, 100 mM NaCl and 1 mM EDTA) and centrifuged at 4000 rpm for 10–15 min, three to five times to remove any excess In(III)-EDTA. After the final wash, the cells were resuspended in 50 ml of the ICP-MS buffer and a 200 µl aliquot was plated in a Corning Costar round bottom polystyrene 96 well plate (catalog number 3799) to measure the OD at 600 nm using a Synergy H1 (BioTek) microplate reader at ambient temperature. The remaining cells were pelleted, frozen, and stored at −80°C until assayed. The day before analysis, the frozen *E. coli* cells were incubated with 2 ml of 35% ICP-MS grade HNO_3_ (AriStar Ultra) overnight at 50°C. Two hundred eighty-six microliter of the digested sample was added to 4.7 ml of deionized water to give final concentration of 2% HNO_3_ and filtered with a 0.22 μm filter. The 35% HNO_3_ that was used for sample preparation was used to prepare a 2% HNO_3_ blank. Internal standard was made in 2% HNO_3_ solution to contain 20 ppb Sc (stock solution: 100 ppm Sc) and 20 ppb 6-Li (stock solution: 100 ppm Sc). Indium(III) standards were made from a purchased stock solution of 1000 ppm In(III) in the range of 0.05–10 ppb. The In(III) signal was measured from the digested lysates diluted 1/100. To correct for signal fluctuations, the 2 ppb and 5 ppb In(III) standards were run between each sample. The raw counts for indium were collected as an average of three readings in five sweeps using an iCAP Q ICP-MS (Thermo Scientific) in the Analytical Laboratory for Environmental Science Research and Training (ANALEST) facility in the Department of Chemistry at the University of Toronto, Toronto, ON. This value was normalized to the obtained OD at 600 nm by assuming that for every 1 OD_600nm_ there are 5 × 10^8^ cells. We assumed an *E. coli* cell volume of 0.7 × 10^−16^ l.

## Results

### Binding of Iron(III)-EDTA to NikA

**Figure 1.**
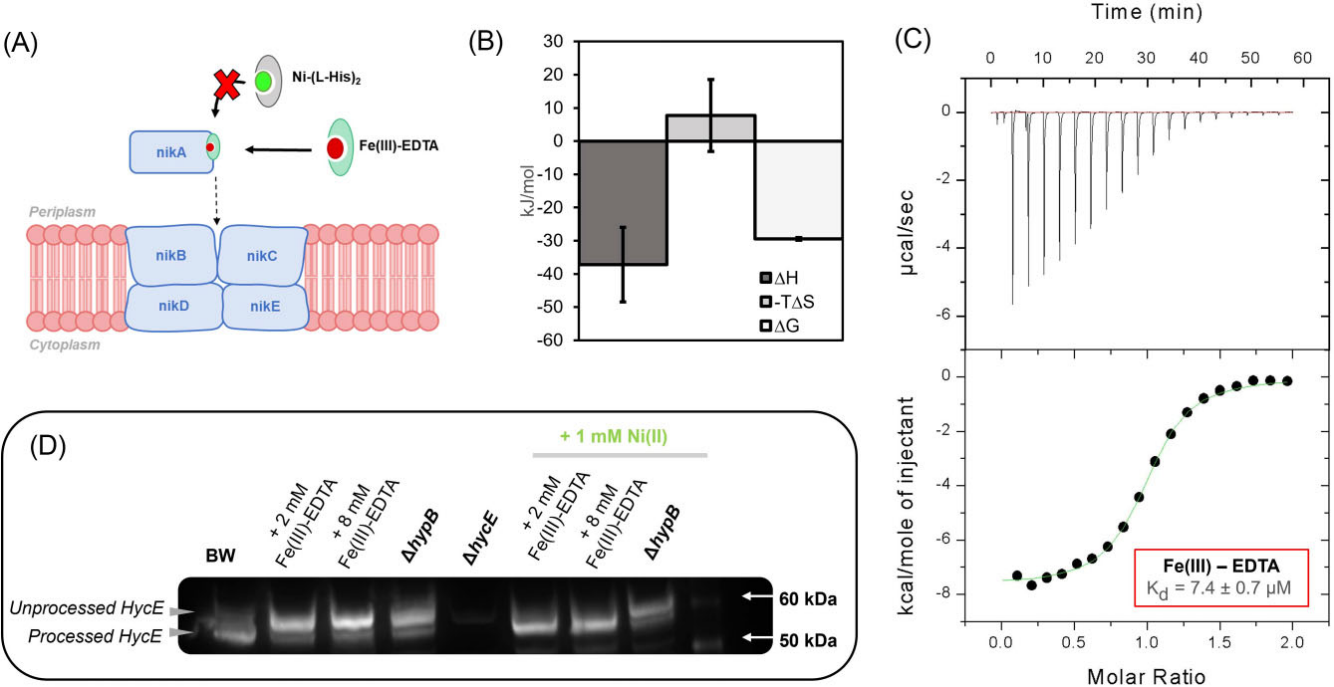
(A) Schematic of hypothesized Fe(III)-EDTA binding to NikA and blocking binding and uptake of the Ni(ii)-(l-His)_2_ ligand. (B) thermodynamic profile of Fe(III)-EDTA binding to NikA. error bars are the standard deviation from all replicate data. (C) A representative isothermal binding curve of Fe(III)-EDTA binding to nika. the top panel conveys the raw data and the bottom panel shows the integrated heat from each injection as a function of the molar ratio of ligand added to protein. (D) A representative immunoblot analysis of the wild type strain (BW1125) grown in the presence of 2 mM or 8 mM Fe(III)-EDTA, with and without the addition of 1 mM nickel. Δ*hypB* strain gives no mature hyce protein. Δ*hycE* knockout strain shows blot specificity.

The binding of metal chelates to *Ec*NikA has been broadly studied in the context of defining the likely biologically relevant chelate, and more recently in the design and reactivity of artificial metalloenzymes [50, 52, 55–59]. From these studies, the highest affinity chelator is the biologically relevant complex Ni-(L-His)_2_ (*K*_d_ = 0.3 uM), however many other chelators have low μM affinity for *Ec*NikA [55]. Fe(III)-EDTA binds *Ec*NikA with a *K*_d_ of 2.5 µM as determined by fluorescence quenching to the nickel binding site (Fig. 1A) [55]. As we hypothesized that other EDTA complexes, that are more stable than Fe(III)-EDTA, may be useful *Ec*NikA inhibitors, we first confirmed the action of Fe(III)-EDTA on [NiFe]-hydrogenase production. Initially, we evaluated the binding of Fe(III)-EDTA to NikA by ITC (*K*_d_ = 7.4 ± 0.7 µM) (Fig. 1C), which gave a binding constant similar to that reported in the literature determined by fluorescence quenching. The thermodynamic profile shown in Fig. 1B is indicative of a similar enthalpy-driven binding mechanism to Ni(II)-(L-His)_2_, with energy values being most similar to the binding of Ni(II)-Gly_2_ and Ni(II)-(L-Asp)_2_ [30]. However it proved impossible to directly measure the effect of the Fe(III)-EDTA complex on [NiFe]-hydrogenase activity in the whole cell using the benzyl viologen assay due to suppression of the colorimetric signal by Fe(III) (Supplementary Fig. 1). Development of the purple color in the hydrogenase assay occurs through the reduction of benzyl viologen dichloride by the hydrogenase enzyme. The reduction potential for benzyl viologen is −347 mV, which is much lower than the reduction potential of Fe(III) to Fe(II), which has been determined to be approximately 740 mV [60, 61]. Thus we hypothesize that Fe(III) is being reduced by electrons produced by [NiFe]-hydrogenase, preventing benzyl viologen reduction. However, using immunoblot analysis of HycE processing, cells treated with Fe(III)-EDTA (2 mM and 8 mM) show an increase in the unprocessed HycE, consistent with intracellular nickel starvation, and that the processing is rescued when excess nickel is added to the media (Fig. 1D). Thus, the experimental evidence confirms that Fe(III)-EDTA binds to NikA and selectively inhibits the [NiFe]-hydrogenase in a cellular context, supporting our inhibition strategy.

### Impacts of other EDTA chelates on [NiFe]-hydrogenase activity

**Figure 2.**
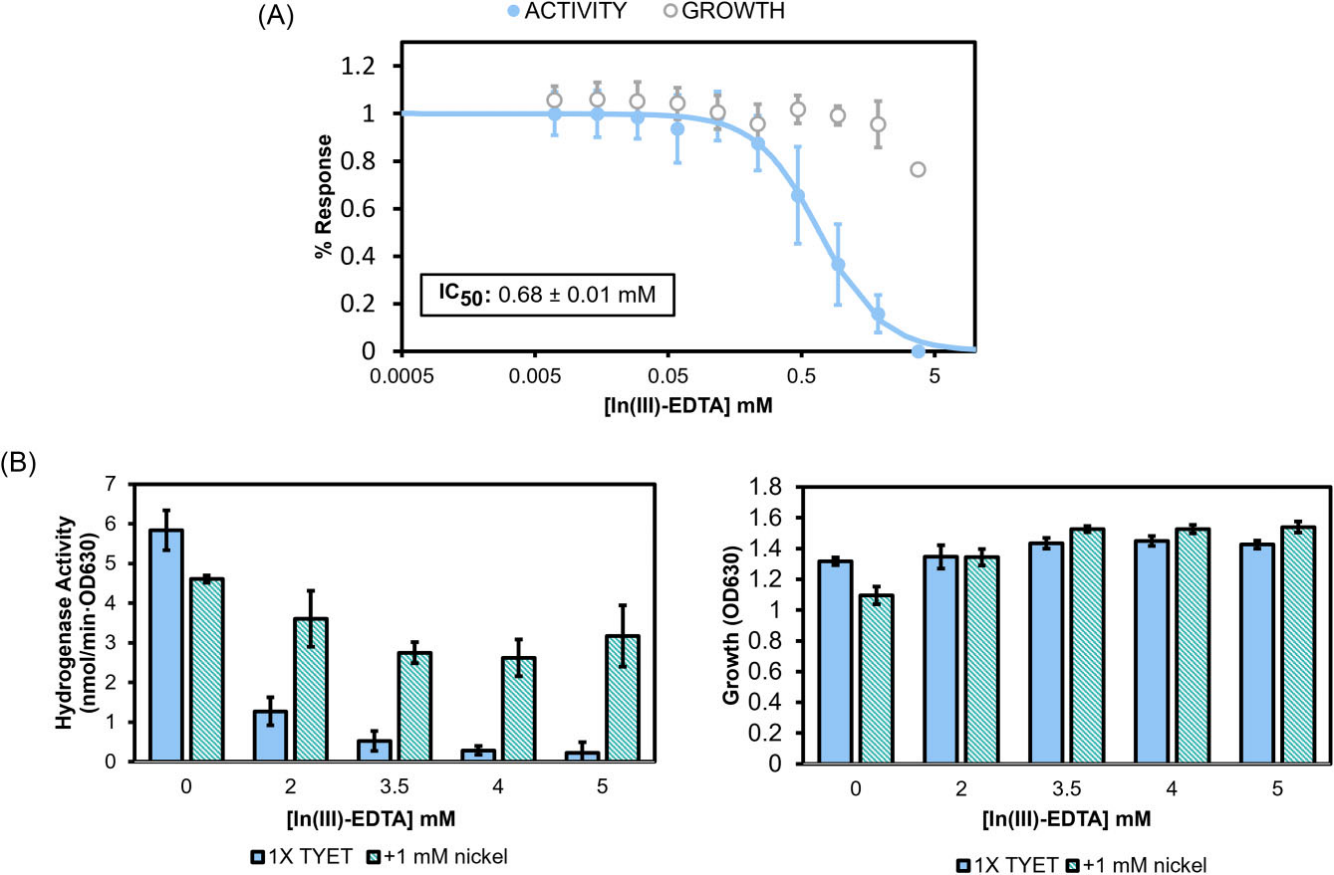
(A) Whole-cell hydrogenase activity (closed circles, blue) and bacterial growth (open circles, gray) in presence of In(III)-EDTA. The activity response was fit to the logistic equation (see experimental). (B) Recovery assay of whole-cell hydrogenase activity in the presence of In(III)-EDTA, without (blue) or with (teal) the addition of 1 mM nickel(II). Whole-cell hydrogenase activity is shown in the chart on the left with complementary growth measured by OD_630_ on the right.

**Figure 3.**
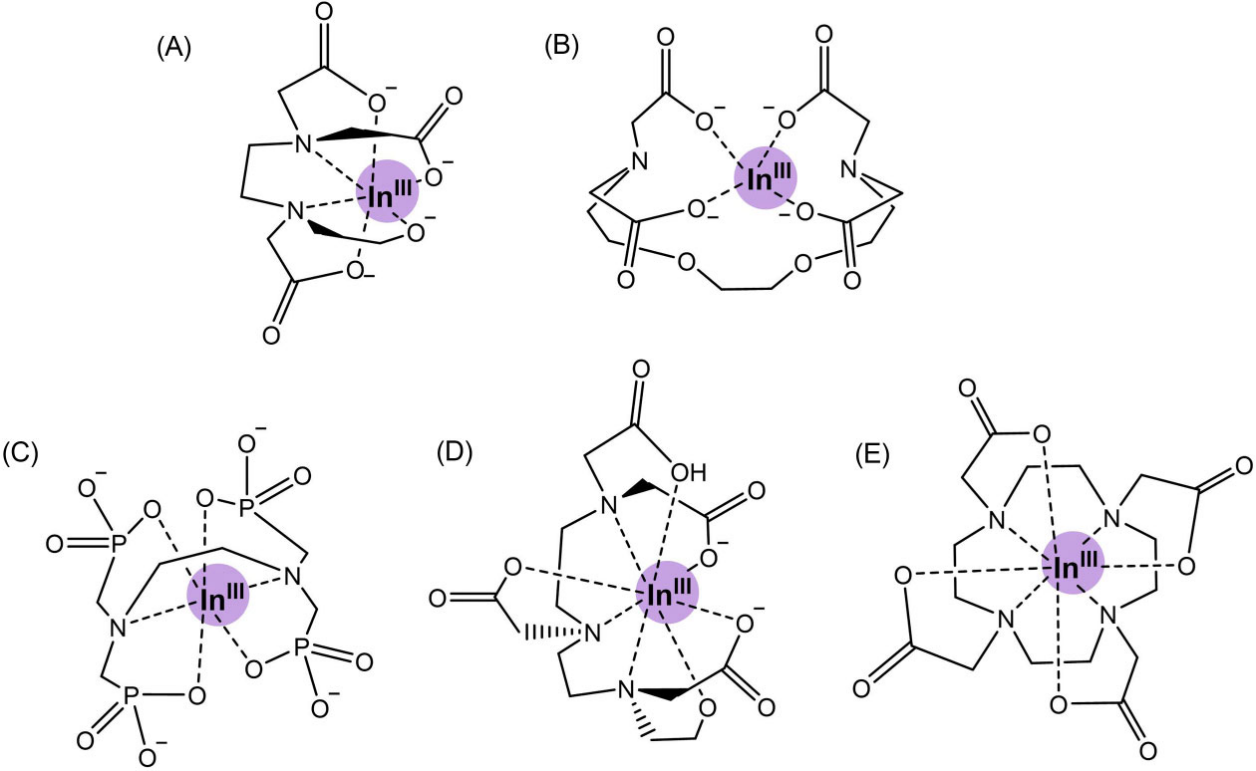
Structures of indium(III) complexed with (A) EDTA, (B) EGTA, (C) EDTPA, (D) DTPA, and (E) DOTA. In(iii)-EGTA and In(iii)-EDTPA depicts hypothesized coordination [67, 68].

Although Fe(III)-EDTA provides promising cell-based inhibition of [NiFe]-hydrogenase activity, it is unlikely to be useful *in vivo*, as Fe(III)-EDTA is rapidly taken up in eukaryotic cells via reduction and likely dissociation of the complex [62]. To replace Fe(III), we evaluated bismuth(III), lutetium(III), and indium(III) which are stable in the same oxidation state and form complexes with EDTA. Bismuth(III) was chosen as its salts have historically been used to treat symptoms caused by stomach-infecting bacteria such as, *H. pylori* [21, 63–65]. Both lutetium and indium were chosen because in biological environments they only exist in the 3+ oxidation state and cannot be readily reduced, therefore would likely not cause signal suppression issues with the whole-cell hydrogenase assay [66]. No interference was observed with In(III)-EDTA in the hydrogenase assay, consistent with the reduction potentials of this species. Unfortunately, the Lu(III)-EDTA formed a precipitate when added to the TYET media used to grow the *E. coli* strains, thus the inhibition of [NiFe]-hydrogenase could not be determined. Using Bi(III)-EDTA, we were able to probe the enzyme maturation through immunoblot analysis (Supplementary Fig. 3), however, there was notable signal suppression in the whole cell hydrogenase assay (Supplementary Fig. 2B). Complexes of In(III)-EDTA did not interfere with the whole cell hydrogenase assay (Supplementary Fig. 2A) and a clear dose response was observed upon increasing the In(III)-EDTA concentrations from 0.005 mM to 5 mM, giving an IC_50_ of 0.68 ± 0.01 mM (Fig. 3A). Importantly, recovery of approximately 70% of the hydrogenase activity was observed with the addition of 1 mM nickel in combination with In(III)-EDTA (2–5 mM) (Fig. 2B, left panel). These results support the hypothesis that Ni(II) uptake is inhibited by In(III)-EDTA as in the presence of excess Ni(II), alternative nonspecific uptake mechanisms are possible [17]. Notably, the growth of the cells is not impacted, even with the addition of high concentrations of the In(III)-EDTA complex, which supports selective inhibition of Ni(II) uptake (Fig. 2B, right panel).

### Indium(III)-EDTA selectivity

**Figure 4.**
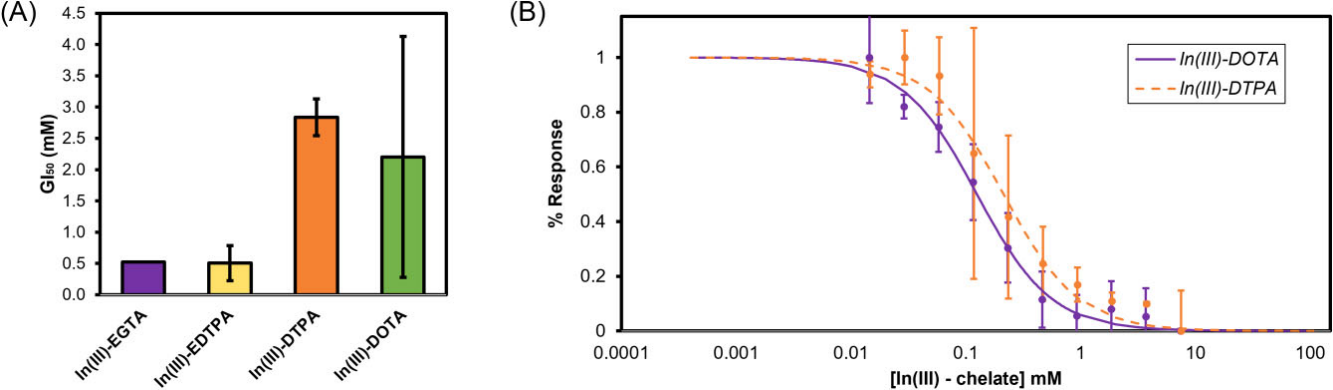
(A) Average of the calculated GI_50_ values in mM from the dose-response curves generated for each indium chelate. (B) Dose response curves of In(III)-DOTA (solid) and In(III)-DTPA (dashed). The activity response was fit to the logistic equation described above of at least three biological replicates and error is shown as normalized standard deviation.

Given the promise of In(III)-EDTA, other In(III) chelates were evaluated. Chelators were chosen based on distinctness of structure and charge (Fig. 3). *E. coli* growth is not inhibited by In(III)-EDTA, however substantial growth inhibition was observed with the other In(III)-chelates (Fig. 4A). Our previous findings revealed that the growth phase of the cells was important to producing reliable assays of hydrogenase activity, thus due to the toxicity of In(III)-EDTPA and In(III)-EGTA, these compounds were not further evaluated (Fig. 3B, C) [17]. For In(III)-DTPA and In(III)-DOTA, bacterial growth varied greatly between replicates, however, these compounds inhibited hydrogenase activity (Fig. 3D, E, Fig. 4B). Due to the varied growth inhibition of the DTPA and DOTA chelates, these complexes were not pursued further (Fig. 4).

### Indium(III)-EDTA binds NikA and impacts [NiFe]-hydrogenase maturation

**Figure 5.**
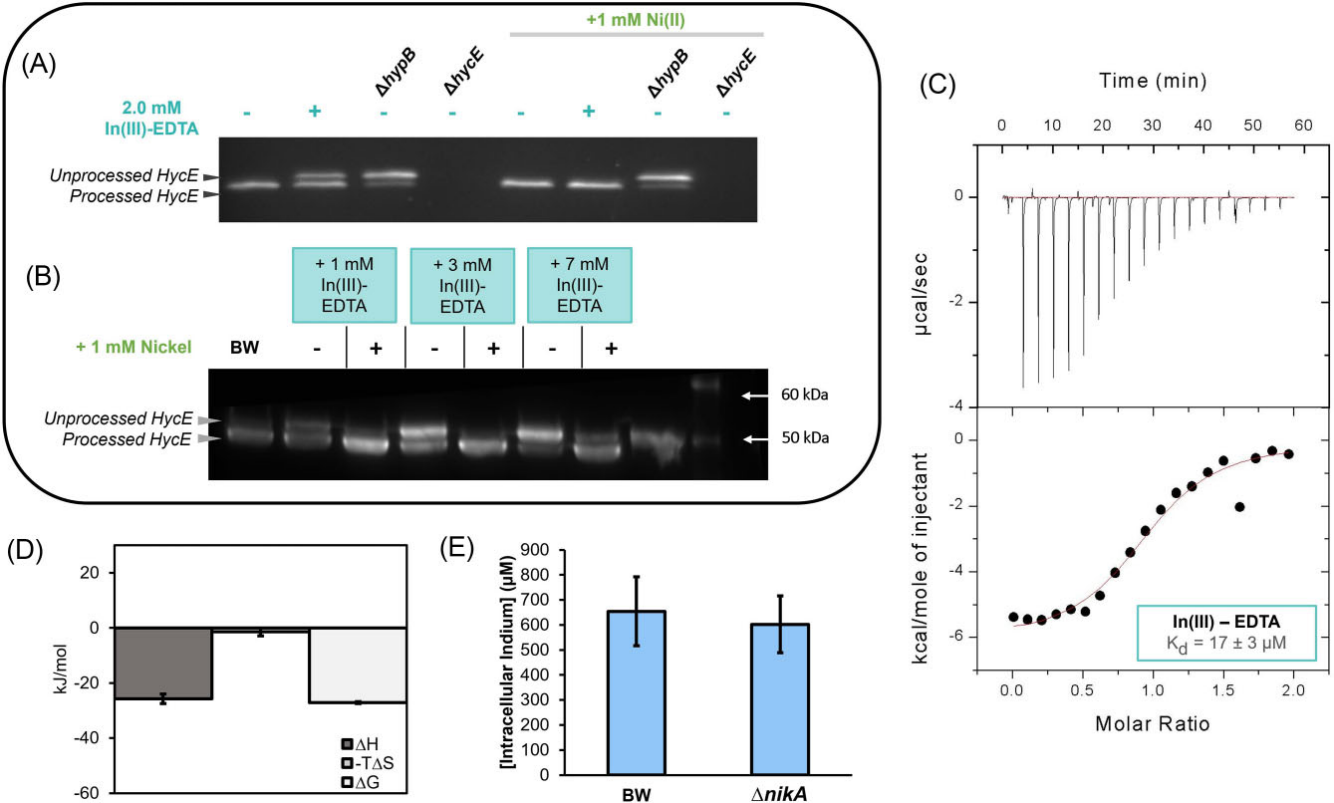
(A) A representative immunoblot of the wild type strain (BW1125) grown in the presence of 2 mM In(III)-EDTA, with and without the addition of 1 mM nickel. Δ*hypB* and Δ*hycE* were knockout strains used as positive and negative controls, respectively. (B) A representative immunoblot of the wild type strain (BW1125) grown in the presence of 1 mM, 3 mM, or 7 mM In(III)-EDTA, with and without the addition of 1 mM nickel. Δ*hypB* and Δ*hycE* were knockout strains used as positive and negative controls, respectively. (C) A representative isothermal binding curve of In(III)-EDTA binding to nika. The top panel conveys the raw data with the bottom panel plotting the heat from each injection as a function of the molar ratio of ligand added to protein. (D) Thermodynamic profile of In(III)-EDTA binding to nika. Error bars are the standard deviation from all replicate data. (E) ICP-MS quantified intracellular indium concentration per cell in wild-type, BW25113 vs Δ*nikA*.

The In(III)-EDTA complex was promising given its limited toxicity on growth, the micromolar inhibition of hydrogenase activity, and the rescue of activity with added nickel. Next, the maturation of HycE was probed through immunoblot analysis to verify that the inhibition is via the [NiFe]-hydrogenase pathway. The representative blot in Fig. 5A shows that with the addition of inhibitory concentrations of In(III)-EDTA (2 mM), there is an emergence of unprocessed HycE consistent with preventing maturation of the Hyd-3 complex (Fig. 5A, B) [17, 29]. The Δ*hypB E. coli* control is included as HycE is not processed in this strain [8, 35]. The Δ*hycE E. coli* knockout strain verifies the α-HycE antibody is binding specifically.

Next, the binding affinity of In(III)-EDTA to NikA was evaluated with ITC. A representative isotherm from three biological replicates is shown in Fig. 5C, giving a binding constant (*K*_d_) of 17 ± 3.0 µM. Analysis of the thermodynamic signature shows that both the enthalpic and entropic components are attenuated in comparison to Fe(III)-EDTA binding to NikA (Fig. 5D). There is very little change in entropy, similar to previous findings with Cu(II)-(L-His)_2_ binding to NikA. Binding of the other indium chelators (In(III)-DOTA, In(III)-DTPA) did not produce binding isotherms, further supporting the selective action of the In(III)-EDTA chelate (Supplementary Fig. 4).

ICP-MS was conducted to determine if In(III) accumulated in the bacteria cells after treatment. It is possible that In(III)-EDTA is transported into the cells via NikA and the Ni(II) transporter. However, comparison of the In(III) uptake in the wild type and the ∆*nikA* strain treated with In(III)-EDTA showed similar levels of indium, suggesting that In(III)-EDTA is not transported via NikA and that the chelate is acting in a competitive fashion for chelate binding to NikA (Fig. 5E), however significant In(III)-EDTA is associating with the cells in a NikA independent fashion [69].

## Discussion

### Targeting NikA and nickel homeostasis

*E. coli* [NiFe]-hydrogenase biosynthesis begins in the periplasm with *Ec*NikA. It is well established that ∆*nikA* strains lack [NiFe]-hydrogenase activity [17, 30, 70]. The activity loss can be recovered with the addition of excess nickel (1 mM) in the whole cell hydrogenase assay, which floods the system with nickel and bypasses the high affinity uptake mechanism [29].

**Figure 6.**
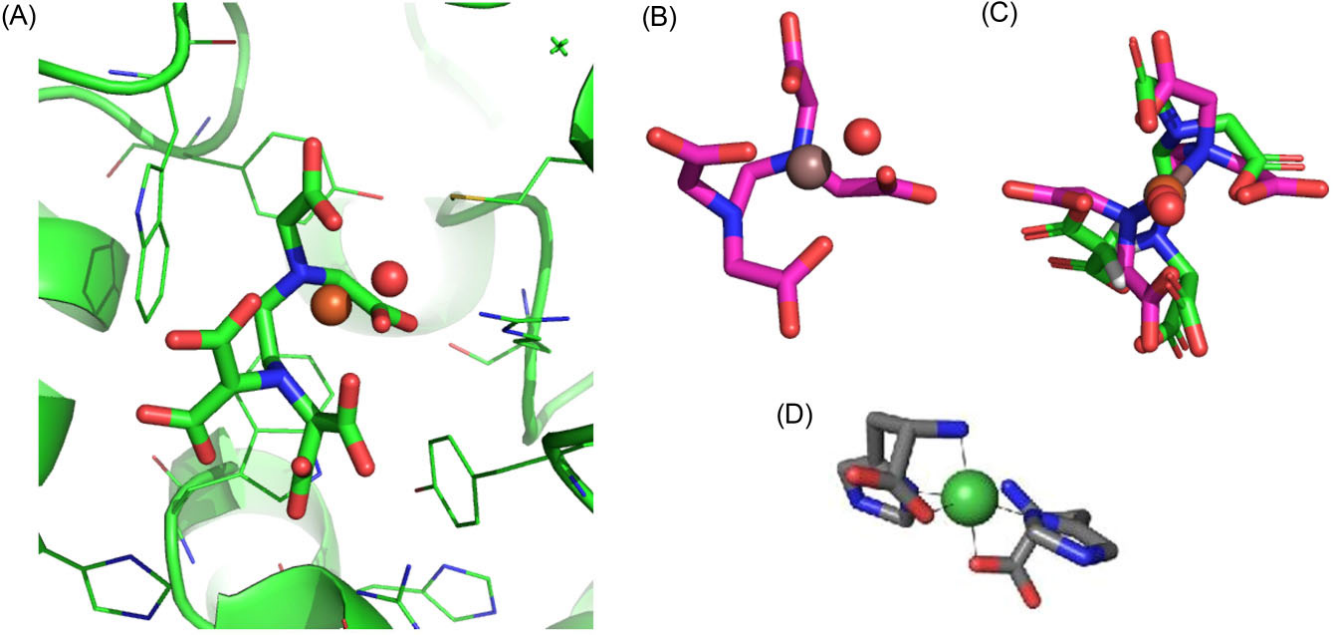
Crystal structures of ligand coordination to (A) Fe(III)-EDTA(H_2_O)^−^, PDB: 1ZLQ, (B) In(III)-EDTA(H_2_O)^−^, and (C) chelate overlay [84].

The biologically relevant Ni(II) chelate ligand of NikA was initially elusive, and complexes of Fe(III)-EDTA and Ni(II)-butane-1,2,4-tricarboxylate were found prior to the identification of the Ni(II)-(L-His)_2_ complex [52, 71]. Other Fe(III)-ethylenediamine based ligand complexes have also been shown to bind NikA with *K*_d_ values in the low µM range [71]. Structural and computational analysis of these NikA-complexes have shown significant plasticity in the NikA-chelate binding with key important salt bridges and dispersive interactions with the ligands alkyl groups driving the interaction [52]. Structure activity relationships of Zn(II)-AMA based chelates have similarly shown that modifications to the ligand are tolerated, however surprisingly, efficacy in inhibiting Ni(II) uptake is not well correlated with NikA chelate affinity [51]. The Fe(III)-EDTA to NikA interaction was validated in this study by ITC (*K*_d_ 7.0 ± 0.7 µM) and we demonstrated this chelate inhibits [NiFe]-hydrogenase maturation, through immunoblot assays. These results support studies to find other chelates for NikA which could be useful for inhibiting the virulence associated with Ni(II)-dependent metalloenzymes in bacteria. Metal complexes have proven useful in targeting other proteins [72], exemplified by the use of copper Schiff bases to inhibit urease in *H. pylori* and gallium-protoporphyrin IX-based targeting of iron uptake [73, 74]. Similarly, copper and iron Schiff bases have been used to successfully inhibit serine proteases (Fig. 1 –6A) [75].

### In(III)-EDTA as a potential antimicrobial

Fe(III)-EDTA has been investigated as an iron supplement due to the facile uptake of the chelate into eukaryotic cells. Although the detailed mechanism of uptake has not been established, the process involves the reduction of Fe(III) to Fe(II) [62]. Likely the reduction is a requirement for the dissociation of iron from EDTA, as the Fe(II) complex is 10^10^ lower in affinity than the Fe(III)-EDTA complex. Based on the binding of Fe(III)-EDTA to *Ec*NikA resulting in the inhibition of [NiFe]—hydrogenase maturation, we hypothesized that metal-EDTA chelates, which are not readily reduced, may be useful as inhibitors of *Ec*NikA in future *in vivo* studies. In(III)-EDTA was a suitable choice, as this chelate has a similar structure to Fe(III)-EDTA, a similar stability constant (Fe log β 25.1, In log β 25.3), and a very low oxidation potential (Fe(III)-Fe(II) 770 mV, In(III)-In(II) ∼−450 mV) [60, 76]. Interestingly, despite the number of NikA metal complexes investigated, complexes beyond Ni(II), Zn(II), Fe(III), and Ru(II) have not been reported [77]. The majority of biological studies of In(III) chelates have focused on the use of indium in radiochemistry due to the auger electron emission of ^111^In and its use as a SPECT/PET imaging agent [77, 78]. The use of ^111^In(III)-EDTA complexes are reported in the context of covalent protein chelate bioconjugates [79, 80]. Reports of using ^113m^In(III)-EDTA as a noninteracting tracer for blood brain barrier permeation studies suggest this complex is quite inert *in vivo* [81]. Other reports of biological effects of In(III) complexes have shown that the activity is highly dependent on the metal ligation [78].

The intestinal microflora also consist of several organisms that use hydrogenases. Introducing In(III)-EDTA as a potential inhibitor of related pathogens could jeopardize the hydrogenase activity of nonpathogens. However, a large majority of the gut microbiota, particularly *Bacteroides* and *Firmicutes*, of which most encode [FeFe]-hydrogenases, there is a lower percentage that encodes the oxygen tolerant [NiFe]-hydrogenases (∼20%) [82]. Our hydrogenase assay probes the formate-dependent hydrogen (HycE) evolution which appears quantitatively lower in the surveyed gut microbiomes compared to the ferredoxin-dependent hydrogen evolution of the dominant [FeFe]-hydrogenases [82]. Overall gut physiology may not be directly impacted by the introduction of In(III)-EDTA, due to HycE not being favored in resident gut bacteria, however changes in nickel availability and homeostasis could lead to dysbiotic effects if a NikA homolog is present [14].

### In(III)-EDTA: mechanism of action

The highest affinity ligand for NikA is its cognate ligand Ni(II)-(L-His)_2_ which has a binding constant in the 0.3–0.8 µM range [30, 83]. However, histidine is required for formation of the complex and nickel uptake which may be limiting in some environments [71]. Sychantha *et al*. investigated the competitive binding of Zn(II)-aspergillomarasmine (Zn(II)-AMA) to NikA from *Klebsiella pneumoniae* and discovered this binding can inhibit urease production and hydrogenase production. Zn(II)-AMA has a *K*_d_ of approximately 2 µM for *Ec*NikA, which is four-fold weaker than Ni(II)-(L-His)_2_. In this study, the binding of In(III)-EDTA was found to be 17 ± 3 µM to *E. coli* NikA, which is eight-fold weaker than Zn(II)-AMA [51]. We observe an approximately two-fold weaker binding with In(III)-EDTA than Fe(III)-EDTA for *Ec*NikA which may be due to the larger size of In(III) over Fe(III). Comparison of the thermodynamic profiles of Fe(III)-EDTA and In(III)-EDTA NikA binding, shows that the In(III) chelate gives attenuated entropy and enthalpy values which sum to give an overall weaker interaction (Fig. 1C, 5C). Figure 6 shows Fe(III)-EDTA bound to NikA (PDB: 1ZLQ) and an overlay of the Fe(III) chelate with the structure of the In(III)-EDTA chelate from aqueous solution [52]. One interpretation of the ITC data and the structural information is that In(III)-EDTA binds NikA in a distorted octahedral geometry, similar to Fe(III)-EDTA, but with suboptimal contacts which may lead to a restriction in the formation of the closed NikA confirmation [30].

Further characterization through the immunoblot assays supports that In(III)-EDTA is affecting the biosynthesis of [NiFe]-hydrogenase. The appearance of the unprocessed HycE and disappearance of the processed mature enzyme indicates that In(III)-EDTA is in fact impacting nickel movement across the membrane. We proposed three hypotheses for the role of In(III)-EDTA through the system: (i) the entire In(III)-EDTA complex is passed along the NikBCDE transporter, and sequestered within the cell potentially in a metallochaperone complex; (ii) the indium alone is passed into the cell and, again is sequestered, or (iii) the In(III)-EDTA bound to NikA is unable to be passed onto the NikBCDE transporter and thus remains bound to NikA. Performing ICP-MS measurements of indium content on wild type (BW25113) *E. coli* and the Δ*nikA* strain showed that there is a negligible difference between the amount of indium per cell. Due to this negligible difference, it is concluded that the complex is not passing through the transporter and accumulating within the cell. This further suggests that there is a competitive mechanism between the In(III)-EDTA and the nickel(II) in the system for binding to NikA.

Our previous work highlighted the importance of considering cell growth when assaying the [NiFe]-hydrogenase pathway [17]. The bioactive small molecule screen presented iodoquinol as a potential “hit” in the assay due to the inhibition of whole cell hydrogenase activity and high levels of unprocessed HycE. However, further studies showed confounding and likely off-target effects of this molecule beyond inhibition of the [NiFe]-hydrogenase activity due to bacterial growth inhibition. In(III)-EDTA is significantly improved in this aspect as no growth inhibition is seen even with the addition of up to 5 mM of In(III)-EDTA (Fig. 2B). Inhibition of cell growth poses a natural selective pressure on a pathogen and facilitates a means to develop resistance mutations [16, 85, 86]. The lack of this selective pressure with In(III)-EDTA proves advantageous and can help us understand [NiFe]-hydrogenase inhibition via NikA.

## Conclusion

Binding metal chelates to NikA is a promising strategy to block the formation of the virulence-related metalloproteins [NiFe]-hydrogenase and urease. We have established that the Fe(III)-EDTA chelate does inhibit [NiFe]-hydrogenase maturation in whole cell assays. Interestingly, the isosteric In(III)-EDTA complex also inhibits the Ni(II) uptake pathways in a similar fashion and is not toxic to *E. coli* growth. This is in contrast to other In(III) chelates with DTPA, DOTA, EGTA, and EDTPA, which likely act by multiple mechanisms causing cell toxicity. Our work suggests that In(III)-EDTA is able to selectively inhibit [NiFe]-hydrogenase activity, and thus further studies should probe the exact mechanisms downstream of NikA, as well as evaluating In(III)-EDTA in an *in vivo* model. Although In(III)-EDTA is unlikely to be a useful antibiotic due to its modest affinity for NikA, the lack of toxicity and observed inhibition of [NiFe]-hydrogenase maturation suggests related chelates with structures optimized for binding NikA may provide a novel antibacterial strategy targeting Ni(II) import and Ni(II)-dependent pathogenicity.

## Supplementary Material

mfaf008_Supplemental_Files

## Data Availability

The data underlying this article will be shared on reasonable request to the corresponding author.
