# Supplementary figures and images for: Selective inhibition of NikA mediated Ni(II) import in E. coli by the Indium(III)-EDTA complex
Source: Metallomics. 2025 Mar 4;17(4):mfaf008. doi: 10.1093/mtomcs/mfaf008 (PMC12086673; doi:10.1093/mtomcs/mfaf008)

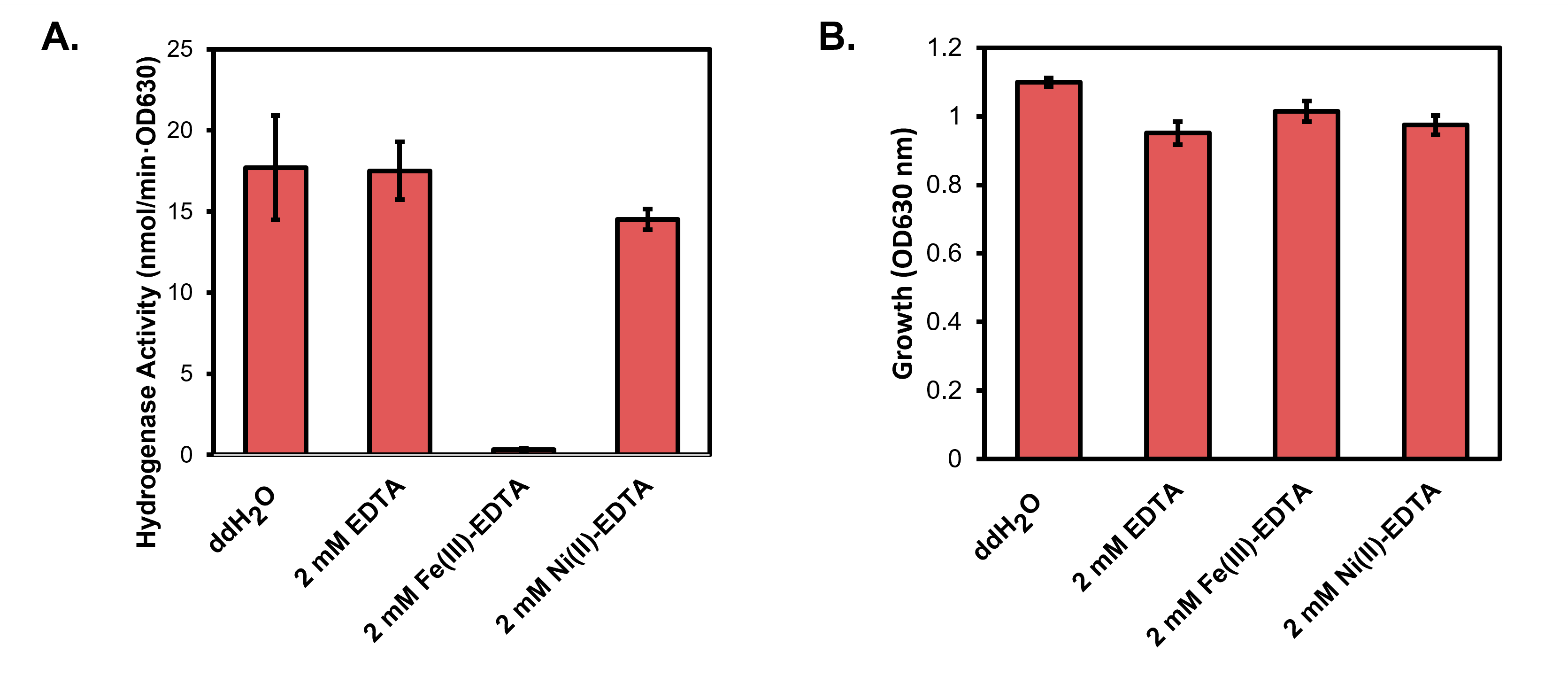

Supplement: mfaf008_Supplemental_Files [file mfaf008_supplemental_files.zip › Suppl_data_Figure_S1.tif]

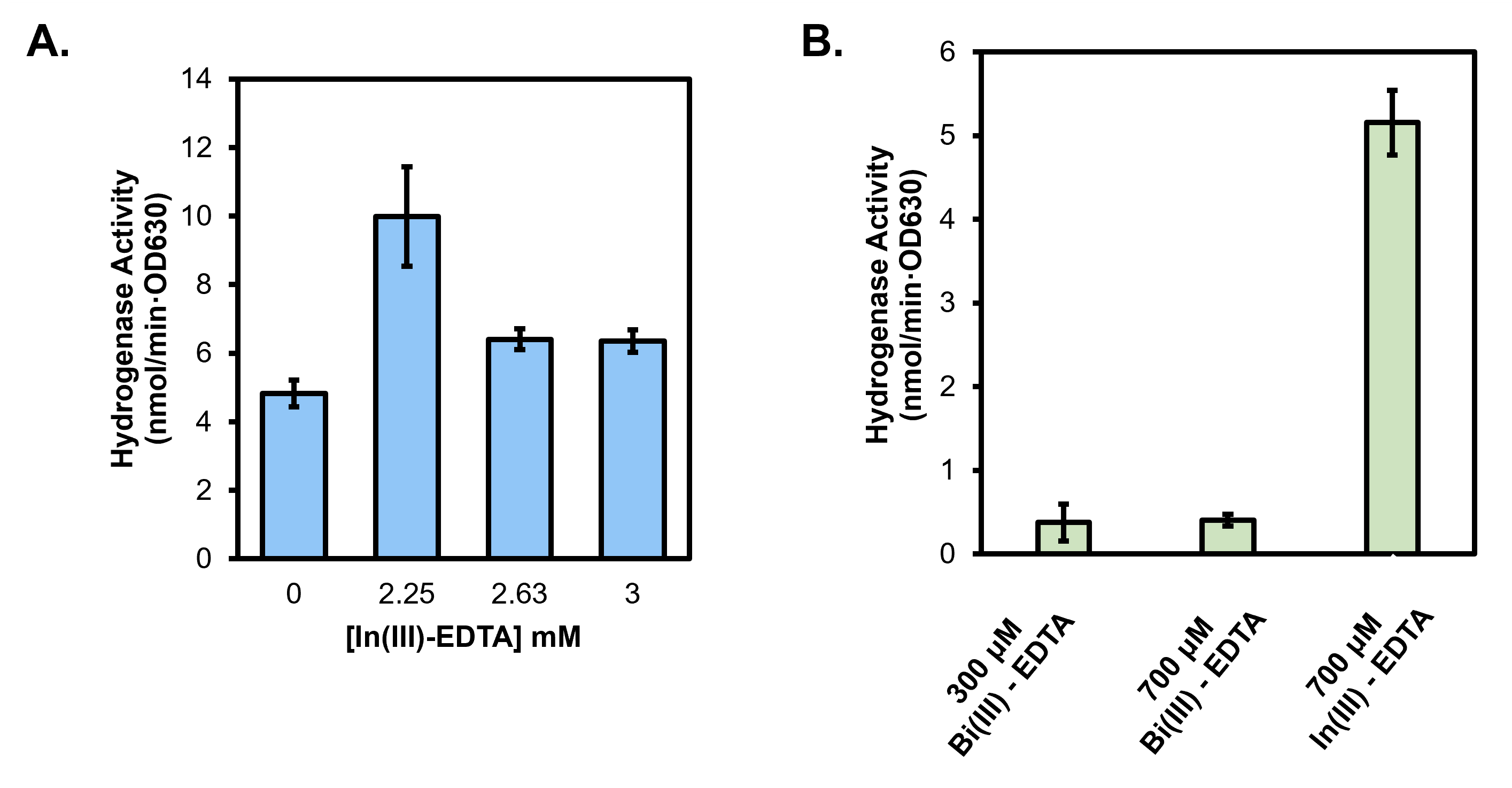

Supplement: mfaf008_Supplemental_Files [file mfaf008_supplemental_files.zip › Suppl_data_Figure_S2.tif]

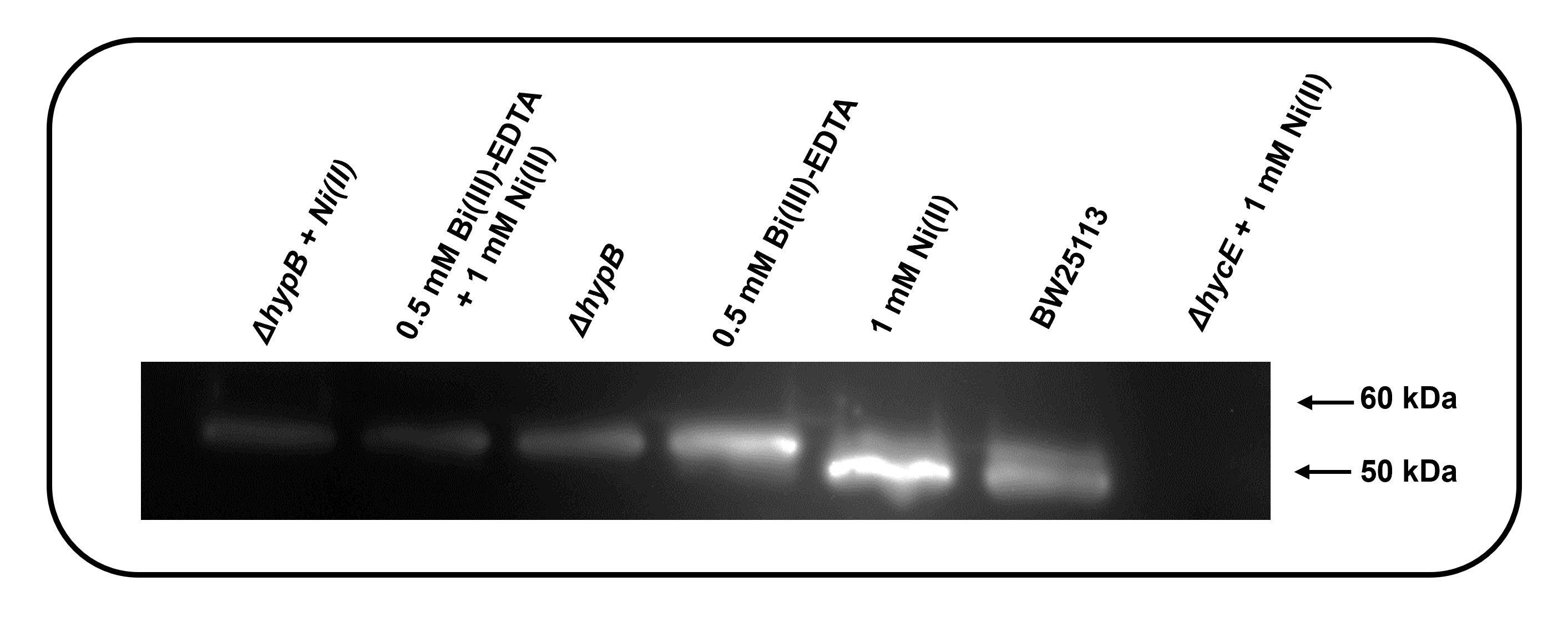

Supplement: mfaf008_Supplemental_Files [file mfaf008_supplemental_files.zip › Suppl_data_Figure_S3.tif]

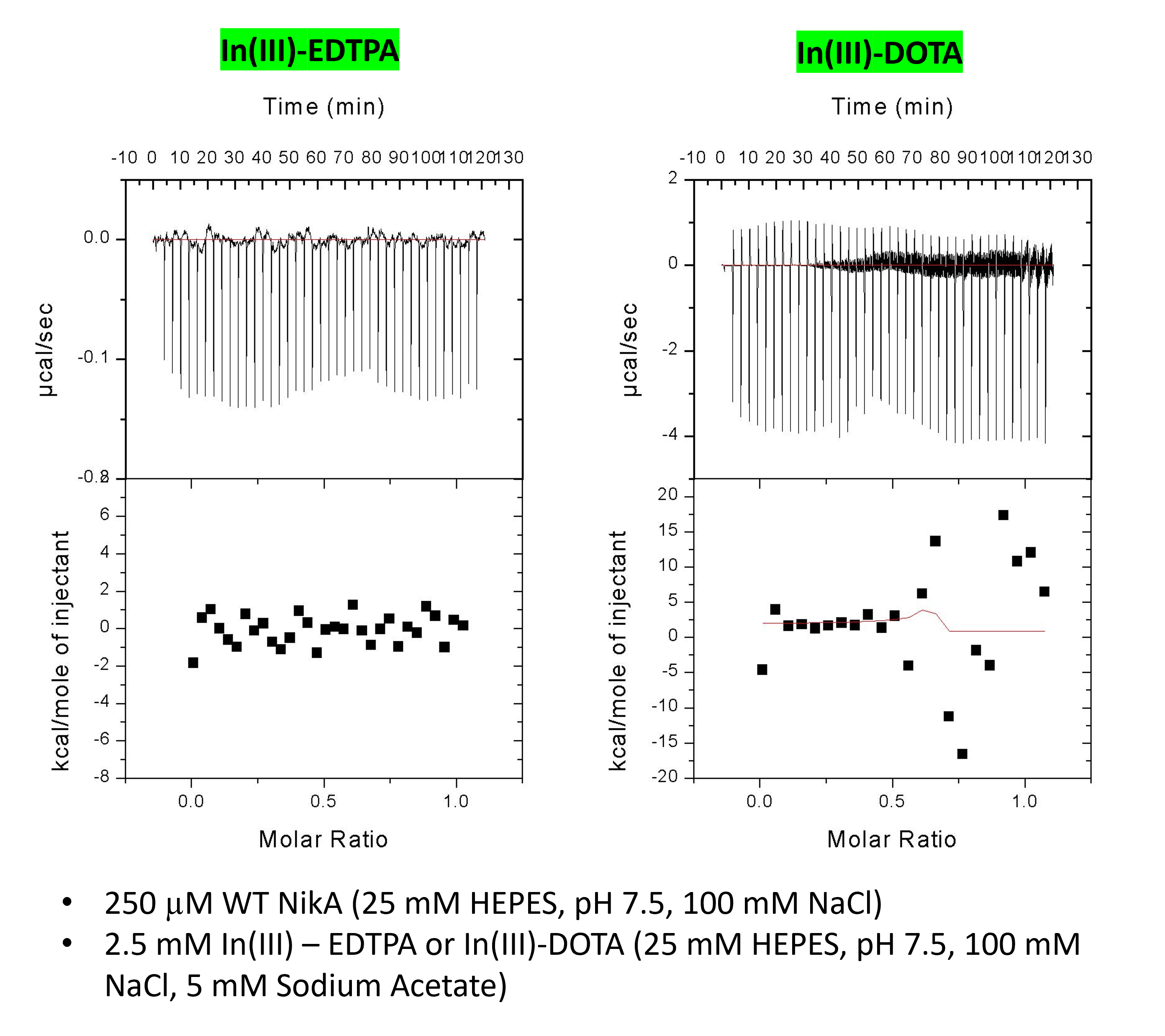

Supplement: mfaf008_Supplemental_Files [file mfaf008_supplemental_files.zip › Suppl_data_Figure_S4.tif]
